# Determinants of Acute Kidney Injury After Endoscopic Retrograde Cholangiopancreatography in Patients With Liver Cirrhosis: Retrospective Observational Study

**DOI:** 10.2196/87551

**Published:** 2026-07-06

**Authors:** Chieh Wei Chang

**Affiliations:** 1 Ditmanson Medical Foundation Chia-Yi Christian Hospital Chiayi City Taiwan

**Keywords:** acute kidney injury, AKI, endoscopic retrograde cholangiopancreatography, ERCP, liver cirrhosis, nationwide inpatient sample, NIS

## Abstract

**Background:**

Patients with cirrhosis may have acute kidney injury (AKI) after receiving endoscopic retrograde cholangiopancreatography (ERCP).

**Objective:**

This study aimed to identify determinants of AKI that require dialysis following ERCP in patients with cirrhosis.

**Methods:**

Data from the US Nationwide Inpatient Sample from 2016 to 2020 were retrospectively reviewed. Patients aged ≥20 years with liver cirrhosis who underwent ERCP were identified. The primary outcome was AKI requiring dialysis. Logistic regression with stepwise selection was used to identify factors associated with dialysis-requiring AKI among demographic variables, comorbidities, and hospital characteristics.

**Results:**

Data from 6748 patients with liver cirrhosis who underwent ERCP were analyzed, and 2.2% (n=148) developed AKI that required dialysis. After adjustment, the results showed that decompensated liver cirrhosis (adjusted odds ratio [aOR] 4.73, 95% CI 3.12-7.15; *P*<.001), chronic kidney disease (aOR 5.93, 95% CI 4.00-8.79; *P*<.001), obesity (aOR 1.65, 95% CI 1.05-2.59; *P*=.03), and sepsis (aOR 3.57, 95% CI 2.44-5.23; *P*<.001) were significant factors associated with AKI that required dialysis. The developed model demonstrated good calibration and discrimination (c-index: 0.826 for derivation cohort, 0.824 for validation cohort).

**Conclusions:**

Decompensated liver cirrhosis, preexisting chronic kidney disease, obesity, and sepsis are significant factors associated with AKI that require dialysis following ERCP in patients with liver cirrhosis. These findings can inform risk stratification and management strategies to improve outcomes in this high-risk population.

## Introduction

Liver cirrhosis is a major cause of morbidity and mortality among individuals with chronic liver disease, and in 2019, it accounted for 2.4% of global deaths [1]. Patients with liver cirrhosis have a 15% to 50% incidence of acute kidney injury (AKI), and for hospitalized patients, the 30-day mortality rate ranges from 30% to 70%, depending on the AKI stage and patient comorbidities [2-4]. Liver disease and renal dysfunction can both result from systemic conditions affecting these organs, but it is more common for primary liver disorders to lead to renal complications [5]. The intricate relations between liver and kidney dysfunction pose a significant clinical challenge, requiring a detailed understanding of the risk factors and predictors to enhance patient outcomes.

Endoscopic retrograde cholangiopancreatography (ERCP) is an advanced endoscopic method used for diagnosing and treating conditions of the biliary and pancreatic systems [6]. The annual number of ERCP procedures has grown consistently, now surpassing 350,000 annually in the United States [7]. ERCP is one of the most technically challenging endoscopic procedures and requires a high degree of knowledge of biliopancreatic anatomy and physiology [8]. Despite decades of improvements, the complication rate of ERCP is still high, with post-ERCP pancreatitis being the most common complication [9]. A recent study has shown that AKI is an important complication after ERCP and is associated with prolonged hospital stays and poor outcomes [10].

Chronic kidney disease (CKD) and end-stage renal disease are known risk factors for complications and in-hospital mortality after ERCP [11,12]. However, data on AKI, particularly dialysis-requiring AKI, following ERCP in patients with liver cirrhosis are limited. This gap in knowledge underscores the need for comprehensive studies to identify specific determinants of AKI that require dialysis in this patient population. Therefore, this study aimed to identify the risk factors for AKI that require dialysis following ERCP in patients with liver cirrhosis using a large-scale, nationally representative dataset.

## Methods

### Study Design and Patient Selection

This retrospective study used data from the US Nationwide Inpatient Sample (NIS) database, managed by the Healthcare Cost and Utilization Project (HCUP) under the US National Institutes of Health. The NIS database collects comprehensive data on approximately 8 million hospital admissions each year, including patient demographics, diagnostic and treatment details, admission and discharge timings, length of stay, and hospital characteristics. Further information about the NIS database is available on the HCUP Agency for Healthcare Research and Quality website.

This study included patients aged ≥20 years who were diagnosed with liver cirrhosis and underwent ERCP between 2016 and 2020. These patients were identified in the NIS database using the *International Classification of Diseases, 10th Edition* (*ICD-10*) codes, and all variables were defined as binary indicators based on physician-diagnosed conditions. Patients with incomplete data on sex and study end points were excluded, as were those with a history of kidney transplantation or those on chronic hemodialysis (with dialysis codes but no AKI code). The ICD codes used for identifying these conditions are listed in Multimedia Appendix 1.

### Ethical Considerations

Data for this research were obtained from the Online HCUP Central Distributor website following the NIS data use agreement through HCUP. This study was a secondary analysis of the NIS database and did not involve direct interaction with patients or the public. As the database contains no patient-identifiable information, ethics approval from our institutional review board and informed consent from participants were not required in accordance with institutional guidelines.

### Study Outcomes

The primary outcome was dialysis-requiring AKI, defined using *ICD-10* diagnostic codes for AKI (N17.x) together with dialysis-related procedure codes (Z49, 5A1D70Z, 5A1D80Z, 5A1D90Z, and 3E1M39Z) recorded during the same hospitalization. As the NIS database does not contain laboratory data (eg, serum creatinine levels), AKI could not be defined using standard clinical criteria and was therefore identified based on diagnostic and procedure codes.

### Study Variables

Data were collected on patient demographics, including age, sex, race or ethnicity, type of admission, weekend admission, insurance status, and decompensated cirrhosis. Decompensated cirrhosis was defined by the presence of *ICD-10* codes indicating major complications of cirrhosis, including hepatic encephalopathy, portal hypertension, hepatorenal syndrome (HRS), and esophageal varices with or without bleeding. Major comorbidities were recorded, including CKD, obesity, diabetes, alcohol or drug abuse, coronary artery disease, atrial fibrillation, congestive heart failure, chronic pulmonary disease, and rheumatic disease. These conditions were treated as binary variables and defined by the presence or absence of the corresponding *ICD-10* diagnostic codes recorded during the index hospitalization. With regard to obesity, it was defined as a binary variable based on *ICD-10* diagnostic codes indicating obesity (eg, E66.x) or documentation consistent with a BMI ≥30 kg/m². The overall burden of comorbidities was assessed using the Charlson Comorbidity Index.

In addition, post-ERCP complications, including sepsis, infection, pancreatitis, hemorrhage, and perforation, were identified. Hospital characteristics such as bed size and hospital location were also collected. Demographic and hospital characteristics were obtained from the NIS database variables. The ICD codes used to define comorbidities and complications are provided in Multimedia Appendix 1.

### Statistical Analysis

The NIS database includes a 20% sample of annual inpatient admissions in the United States; thus, the SURVEY procedure in SAS software (SAS Institute) was used. Descriptive statistics were presented as number (n) and weighted percentage (%), or as mean (SE). We randomly partitioned the study cohort into a derivation cohort (5079/6748, 75%) and a validation cohort (1669/6748, 25%). The derivation cohort was used to develop a multivariable logistic regression model, while the validation cohort was used for model evaluation.

Univariate logistic regression analysis was used to determine associations between the study variables and the occurrence of AKI that required dialysis after ERCP. The final model was determined using backward selection. All variables examined in the univariate analysis were initially entered into the multivariable logistic regression model, except for rheumatic disease and post-ERCP hemorrhage, which were not included because no AKI events occurred. Backward stepwise selection was then applied to sequentially remove variables until all remaining variables met the prespecified significance level (*P*<.05). A forest plot for the multivariable logistic regression model was generated for model visualization. Model discrimination was assessed using area under the curve (AUC), and calibration was evaluated by comparing predicted and observed probabilities using calibration plots in the derivation cohort and the validation cohort. All *P* values were 2-sided, and *P*<.05 was considered statistically significant. All analyses accounted for the NIS’s complex survey design to ensure accurate national estimates. All statistical analyses were performed using SAS software (version 9.4; SAS Institute). Forest plots were generated using R (version 4.4.1; R Foundation for Statistical Computing; R project website) with the grid, checkmate, abind, forestplot, and dplyr packages.

## Results

### Study Population Selection

**Figure 1:**
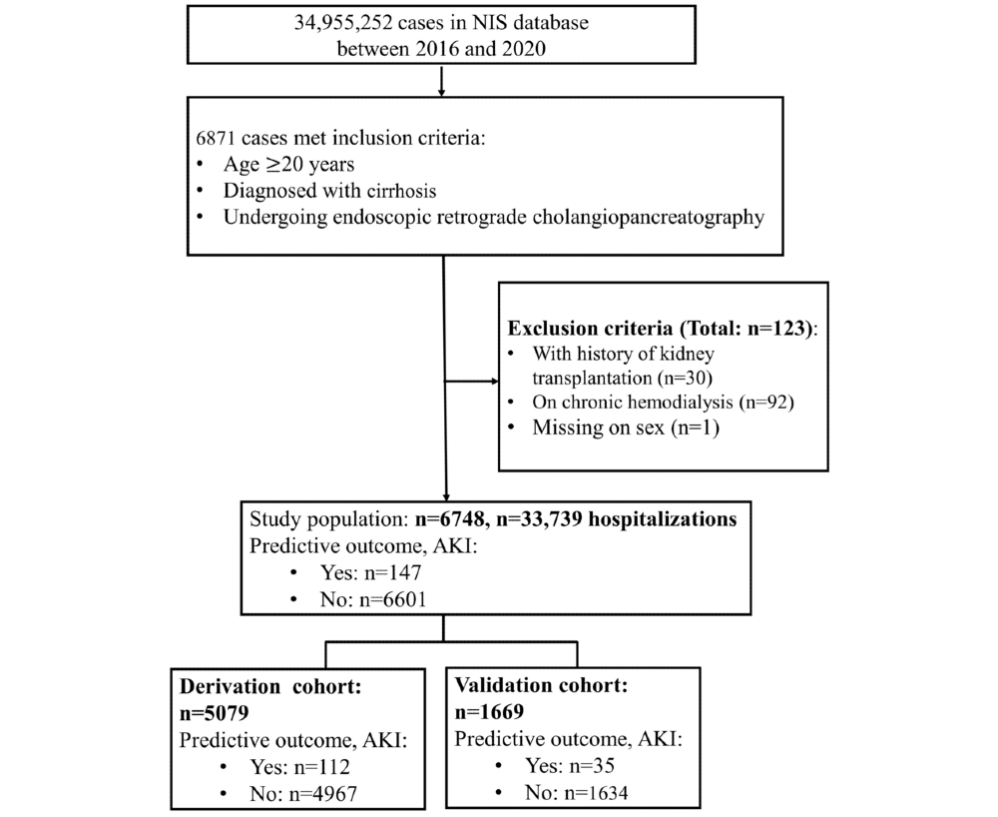
Flowchart of study population selection and allocation. AKI: acute kidney injury; NIS: Nationwide Inpatient Sample.

The process of selecting the study population is illustrated in Figure 1. A total of 6871 patients aged ≥20 years with liver cirrhosis and who underwent ERCP from 2016 to 2020 were identified in the NIS database. Patients who had a history of kidney transplantation (n=30), were on chronic hemodialysis (n=30), and had missing sex information (n=1) were excluded. Thus, the final study sample included 6748 patients, of whom 147 developed dialysis-requiring AKI. After applying discharge weights from the NIS database, this corresponded to a national estimate of 33,739 hospitalizations.

### Characteristics of the Study Population

The characteristics of the patients are described in Table 1. The mean age of the patients was 62.5 (SD 0.18) years, and 2702 (40%) were female. Most of the patients were White (4314/6748; 65.9%), and 2517 (37%) of the patients had decompensated cirrhosis. The patients were randomly divided into the derivation cohort (n=5079) and the validation cohort (n=1669). The data distributions of the 2 groups were similar. In the derivation and validation cohorts, 112 and 35 patients, respectively, developed dialysis-requiring AKI.

**Table 1:** Characteristics of the study populationa.

Characteristics			Overall cohort (N=6748)		Derivation cohort (n=5079)		Validation cohort (n=1669)
**Age group (years), n (%)**							
	20-49	1079 (16)		826 (16.3)		253 (15.2)	
	50-59	1499 (22.2)		1152 (22.7)		347 (20.8)	
	60-69	2123 (31.5)		1575 (31)		548 (32.8)	
	>70	2047 (30.3)		1526 (30)		521 (31.2)	
**Sex, n (%)**							
	Male	4046 (60)		3050 (60.1)		996 (59.7)	
	Female	2702 (40)		2029 (39.9)		673 (40.3)	
**Race or ethnicity, n (%)**							
	White	4314 (65.9)		3262 (66.2)		1052 (65.1)	
	Black	639 (9.8)		470 (9.5)		169 (10.5)	
	Hispanic	1064 (16.3)		800 (16.2)		264 (16.3)	
	Other	529 (8.1)		398 (8.1)		131 (8.1)	
	Missing	202 (2.9)		149 (2.9)		53 (3.2)	
**Insurance status, n (%)**							
	Medicare or Medicaid	4747 (70.5)		3571 (70.5)		1176 (70.5)	
	Private including HMO^b^	1601 (23.8)		1204 (23.8)		397 (23.8)	
	Self-pay, no charge, or other	384 (5.7)		290 (5.7)		94 (5.6)	
	Missing	16 (0.2)		14 (0.3)		2 (0.1)	
**Type of admission, n (%)**							
	Emergent or urgent	6253 (92.8)		4682 (92.3)		1571 (94.4)	
	Elective	484 (7.2)		391 (7.7)		93 (5.6)	
	Missing	11 (0.2)		6 (0.1)		5 (0.3)	
Decompensated cirrhosis, n (%)			2517 (37.3)		1918 (37.8)		599 (35.9)
**Comorbidity, n (%)**							
	CKD^c^	1140 (16.9)		845 (16.6)		295 (17.7)	
	Obesity	1214 (18)		908 (17.9)		306 (18.3)	
	Alcohol or drug abuse	1571 (23.3)		1174 (23.1)		397 (23.8)	
	Diabetes mellitus	2316 (34.3)		1736 (34.2)		580 (34.8)	
	Coronary artery disease	1175 (17.4)		866 (17.1)		309 (18.5)	
	Atrial fibrillation	542 (8)		419 (8.2)		123 (7.4)	
	Congestive heart failure	914 (13.5)		682 (13.4)		232 (13.9)	
	Chronic pulmonary disease	1268 (18.8)		967 (19)		301 (18)	
	Rheumatic disease	171 (2.5)		137 (2.7)		34 (2)	
**CCI^d^, n (%)**							
	0	1897 (28.1)		1452 (28.6)		445 (26.7)	
	1	1429 (21.2)		1071 (21.1)		358 (21.4)	
	2	1220 (18.1)		916 (18)		304 (18.2)	
	>3	2202 (32.6)		1640 (32.3)		562 (33.7)	
**Post-ERCP^e^ complication, n (%)**							
	Sepsis	1808 (26.8)		1356 (26.7)		452 (27.1)	
	Infection	3251 (48.2)		2441 (48.1)		810 (48.5)	
	Post-ERCP pancreatitis	1220 (18.1)		944 (18.6)		276 (16.5)	
	Post-ERCP hemorrhage	9 (0.1)		5 (0.1)		4 (0.2)	
	Perforation	55 (0.8)		41 (0.8)		14 (0.8)	
**Weekend admission, n (%)**							
	No	5196 (77)		3897 (76.7)		1299 (77.8)	
	Yes	1552 (23)		1182 (23.3)		370 (22.2)	
**Hospital bed size, n (%)**							
	Small	769 (11.4)		577 (11.4)		192 (11.5)	
	Medium	1598 (23.7)		1209 (23.8)		389 (23.3)	
	Large	4381 (64.9)		3293 (64.8)		1088 (65.2)	
**Hospital region, n (%)**							
	Northeast	1118 (16.6)		846 (16.7)		272 (16.3)	
	South	1496 (22.2)		1110 (21.9)		386 (23.1)	
	Midwest	2417 (35.8)		1837 (36.2)		580 (34.8)	
	West	1717 (25.4)		1286 (25.3)		431 (25.8)	

^a^Categorical variables are presented as unweighted counts (weighted percentage). Percentages are calculated within each subgroup.

^b^HMO: health maintenance organization.

^c^CKD: chronic kidney disease.

^d^CCI: Charlson Comorbidity Index.

^e^ERCP: endoscopic retrograde cholangiopancreatography.

### Associations Between the Study Variables and the Outcomes

The results of the univariate analysis of the associations between the demographic and clinical characteristics and AKI requiring dialysis are summarized in Table 2. The results showed that insurance status, decompensated cirrhosis, CKD, obesity, congestive heart failure, and post-ERCP complications such as sepsis, infection, and pancreatitis, were associated with AKI that required dialysis. The top 3 conditions with the highest risk for AKI that required dialysis were CKD (odds ratio [OR] 5.5, 95% CI 3.76-8.04), decompensated cirrhosis (OR 4.07, 95% CI 2.74-6.05), and sepsis (OR 3.39, 95% CI 2.35-4.90).

**Table 2:** Associations between the study variables and dialysis-requiring acute kidney injury (AKI) after endoscopic retrograde cholangiopancreatography (ERCP)a.

Study variables		Subgroup, n	Dialysis-requiring AKI, n (%)	OR^b^ (95% CI)	**P* value*
**Age (years), n (%)**					
	20-49	826	23 (2.8)	Reference	—^c^
	50-59	1152	22 (1.9)	0.68 (0.39-1.18)	.17
	60-69	1575	38 (2.4)	0.86 (0.51-1.45)	.58
	>70	1526	29 (1.9)	0.68 (0.40-1.15)	.15
**Sex**					
	Male	3050	73 (2.4)	1.25 (0.86-1.83)	.25
	Female	2029	39 (1.9)	Reference	—
**Race or ethnicity, n (%)**					
	White	3262	79 (2.4)	Reference	—
	Black	470	10 (2.1)	0.88 (0.45-1.70)	.69
	Hispanic	800	13 (1.6)	0.67 (0.35-1.27)	.22
	Other	398	7 (1.8)	0.72 (0.33-1.56)	.41
**Insurance status, n (%)**					
	Medicare or Medicaid	3571	68 (1.9)	Reference	—
	Private, including HMO^d^	1204	38 (3.2)	1.68 (1.13-2.50)	*.01* ^e^
	Self-pay, no charge, or other	290	6 (2.1)	1.09 (0.48, 2.49)	.84
**Type of admission, n (%)**					
	Emergent or urgent	4682	105 (2.2)	Reference	—
	Elective	391	7 (1.8)	0.79 (0.37-1.71)	.56
Decompensated cirrhosis, n (%)		1918	79 (4.1)	4.07 (2.74-6.05)	*<.001*
**Comorbidity, n (%)**					
	CKD^f^	845	57 (6.7)	5.50 (3.76-8.04)	*<.001*
	Obesity	908	28 (3.1)	1.55 (1.01-2.38)	*.045*
	Alcohol or drug abuse	1174	20 (1.7)	0.72 (0.44-1.16)	.18
	Diabetes mellitus	1736	35 (2.0)	0.87 (0.59-1.28)	.49
	Coronary artery disease	866	26 (3.0)	1.49 (0.95-2.33)	.08
	Atrial fibrillation	419	7 (1.7)	0.74 (0.34-1.61)	.45
	Congestive heart failure	682	26 (3.8)	1.99 (1.27-3.12)	*.003*
	Chronic pulmonary disease	967	16 (1.7)	0.70 (0.41-1.20)	.20
	Rheumatic disease	137	0	—	—
**CCI^g^, n (%)**					
	0	1452	18 (1.2)	Reference	—
	1	1071	11 (1.0)	0.83 (0.38-1.79)	.63
	2	916	27 (2.9)	2.42 (1.30-4.50)	*.005*
	>3	1640	56 (3.4)	2.82 (1.61-4.94)	*<.001*
**Post-ERCP complication, n (%)**					
	Sepsis	1356	61 (4.5)	3.39 (2.35-4.90)	*<.001*
	Infection	2441	75 (3.1)	2.23 (1.51-3.30)	*<.001*
	Post-ERCP pancreatitis	944	12 (1.3)	0.52 (0.29-0.92)	*.02*
	Post-ERCP hemorrhage	5	0	—	—
	Perforation	41	2 (4.9)	2.30 (0.55-9.59)	.25
**Weekend admission, n (%)**					
	No	3897	85 (2.2)	Reference	—
	Yes	1182	27 (2.3)	1.05 (0.69-1.59)	.82
**Hospital bed size, n (%)**					
	Small	577	10 (1.7)	0.70 (0.37-1.31)	.27
	Medium	1209	21 (1.7)	0.70 (0.42-1.16)	.17
	Large	3293	81 (2.5)	Reference	—
**Hospital region, n (%)**					
	Northeast	846	18 (2.1)	Reference	—
	South	1110	23 (2.1)	0.97 (0.52-1.83)	.93
	Midwest	1837	37 (2.0)	0.95 (0.53-1.68)	.85
	West	1286	34 (2.6)	1.25 (0.65-2.38)	.499

^a^Categorical variables are presented as unweighted counts (weighted percentage).

^b^OR: odds ratio.

^c^Not applicable.

^d^HMO: health maintenance organization.

^e^*P*<.05.

^f^CKD: chronic kidney disease.

^g^CCI: Charlson Comorbidity Index.

### Factors Associated With AKI Requiring Dialysis After ERCP

**Figure 2:**
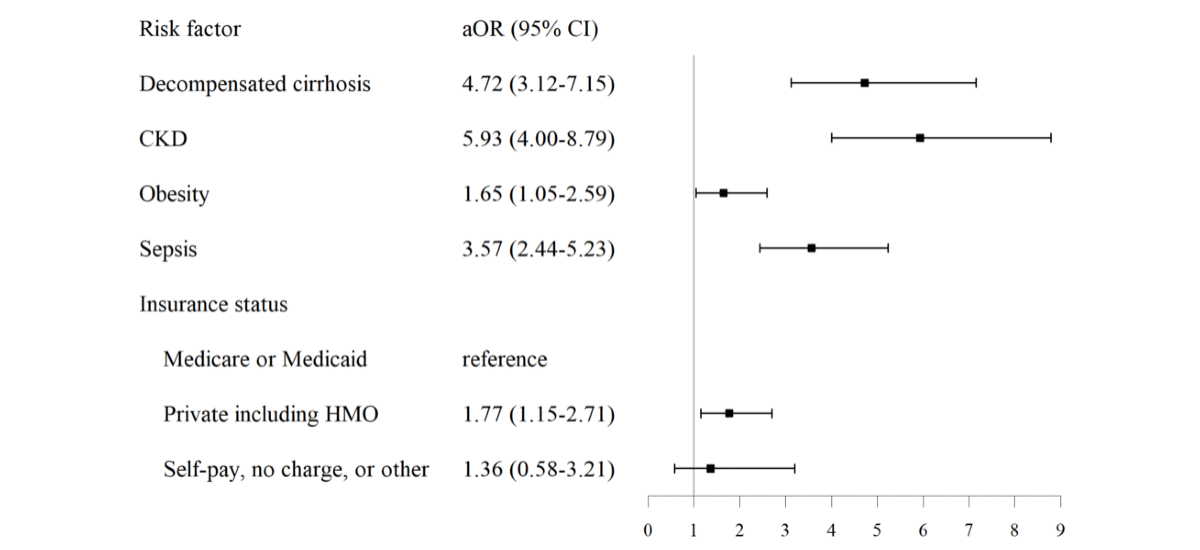
Associations between demographic and clinical characteristics and the risk of acute kidney injury requiring dialysis. aOR: adjusted odds ratio; CKD: chronic kidney disease; HMO: health maintenance organization.

Factors associated with AKI requiring dialysis after ERCP, after adjustment for confounders in the multivariable model, are presented in the forest plot shown in Figure 2. Independent factors associated with AKI that requires dialysis after ERCP were decompensated cirrhosis (adjusted OR [aOR] 4.73, 95% CI 3.12-7.15), CKD (aOR 5.93, 95% CI 4.00-8.79), obesity (aOR 1.65, 95% CI 1.05-2.59), and sepsis (aOR 3.57, 95% CI 2.44-5.23). Notably, insurance status (private including Health Maintenance Organization vs Medicare or Medicaid) was also independently associated with AKI that required dialysis after ERCP (aOR 1.77, 95% CI 1.15-2.71).

**Figure 3:**
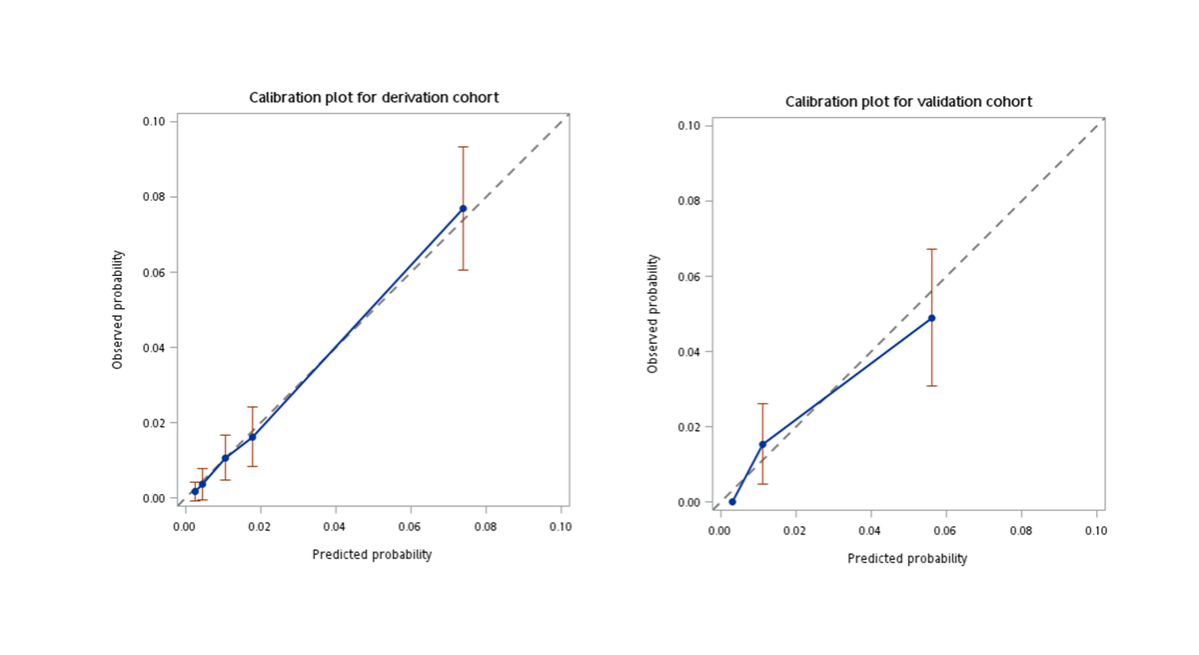
Calibration plots of the model for the derivation cohort and validation cohort. Patients were grouped into 5 and 3 risk groups in the derivation and validation cohorts, respectively, based on predicted probabilities. Points represent observed event rates within each group, with error bars indicating 95% CIs. The dashed line represents perfect calibration.

The model fit was shown to be good in the derivation cohort (AUC=0.825) and in the validation cohort (AUC=0.824). The calibration plots are shown in Figure 3.

## Discussion

### Principal Findings

This study identified significant factors associated with AKI that requires dialysis in patients with liver cirrhosis after ERCP. The model performed well in both the derivation and validation cohorts. Accordingly, the key risk factors were decompensated cirrhosis, CKD, obesity, and sepsis. CKD and decompensated cirrhosis had the highest ORs, indicating strong associations with AKI that requires dialysis. These findings can inform clinical decisions and risk stratification to improve patient outcomes following ERCP in patients with liver cirrhosis.

Liver cirrhosis is a chronic and serious health condition and can progress to the point where the liver is no longer functional [13]. In compensated liver cirrhosis, damage to the liver is less severe and the liver is able to compensate for the damage and continue to function normally but with a reduced level of function [13]. In decompensated liver cirrhosis, the organ is no longer able to perform its normal functions because of severe damage and is characterized by the development of ascites, variceal hemorrhage, and hepatic encephalopathy. The only definitive treatment for decompensated cirrhosis is liver transplantation [13]. Damage to the kidneys is usually present in the natural progression of liver cirrhosis, and HRS is the kidney dysfunction that occurs during the progression of cirrhosis and is characterized by marked impairment of kidney function due to circulatory and hemodynamic alteration that occur with cirrhosis and a heightened systemic inflammatory response [14].

ERCP is a minimally invasive and generally safe procedure when performed by an experienced operator. As with all procedures that use iodinated contrast media, there is a risk that the medium may result in AKI in a small fraction of the patients [9]. Liver cirrhosis, especially decompensated cirrhosis, is associated with increased complications with almost all medical and surgical procedures, and many studies have compared outcomes of ERCP in patients with and without cirrhosis, and the risk of complications in patients with cirrhosis is up to 3-fold greater than that in those without cirrhosis [15,16]. The study by Eyyupkoca et al [17] reported that, compared with patients without liver cirrhosis, those with liver cirrhosis had an increased risk of pancreatitis and cholangitis and that elevated total bilirubin and a higher Child-Pugh class were risk factors for these complications. The study by Bernshteyn et al [18] also reported that Child-Pugh class C was associated with a significantly higher risk of complications after ERCP compared to class A or class B. Recent meta-analyses [19,20] have also shown that compared to patients without liver cirrhosis, those with liver cirrhosis have a significantly higher risk of adverse events including hemorrhage, pancreatitis, and cholangitis.

The study by Solanki et al [21] also reported that in patients with liver cirrhosis, therapeutic ERCP was associated with increased risk of pancreatitis and hemorrhage, whereas diagnostic ERCP was associated with increased risk of pancreatitis and cholecystitis. The authors also noted that comorbidities in patients with liver cirrhosis may increase the risk of post-ERCP complications and mortality. A matched-cohort multicenter study of patients with liver cirrhosis undergoing ERCP reported that liver cirrhosis was associated with an overall increased risk of adverse events, and most notably an increased risk of acute-on-chronic liver failure [22].

As previously noted, kidney dysfunction is part of the natural course of liver cirrhosis, and approximately 20% of patients with cirrhosis experience AKI during the course of the disease [3]. AKI in patients with liver cirrhosis is generally categorized as HRS-associated AKI and non-HRS AKI, and studies have shown that they are distinct clinical conditions with different pathophysiology [2,23]. A recent report by Gadalean et al [10] found that the rate of AKI after ERCP in general (noncirrhotic) patients was 26% and was significantly associated with longer hospital stay and increased in-hospital mortality. Most notably, moderate to severe AKI was associated with a more than 6-fold increased risk of mortality.

Few studies have evaluated the incidence of AKI in patients with liver cirrhosis undergoing ERCP, and none to our knowledge have examined risk factors for AKI that require dialysis.

Nevertheless, a recent study examined the incidence and risk factors of AKI in patients with liver cirrhosis by analyzing the data of over 5 million outpatients, inpatients, and patients admitted to an intensive care unit [24]. The results showed that in hospitalized patients who experienced any decompensation, the rate of AKI was 29% and in stable outpatients it was 28% during a 1-year follow-up period. Infection or sepsis, hepatic encephalopathy, and intensive care unit admission were associated with AKI rates>40%.

The study by Jagtap et al [25] investigated the outcomes of ERCP in patients with decompensated cirrhosis and found higher overall adverse event rates, with cholangitis being significantly associated with an increased risk of mortality. Our results showed that patients with liver cirrhosis and obesity were at increased risk of AKI after ERCP. While we did not identify any studies focusing on obesity and liver cirrhosis in patients undergoing ERCP, the study by Abdelfatah et al [26] reported that neither obesity nor low body weight was associated with pancreatitis following ERCP. The study, however, did not examine AKI. Our results showed that sepsis was a risk factor for post-ERCP AKI in patients with liver cirrhosis. This finding is not unexpected as studies have shown that sepsis is one of the main causative factors of AKI in patients with liver cirrhosis [27,28].

Taken together, patients with liver cirrhosis undergoing ERCP are at a markedly increased risk of complications, and in particular decompensated cirrhosis, CKD, obesity, and sepsis are associated with an increased risk of AKI that requires dialysis. Knowledge of these risk factors can be incorporated into perioperative management and may improve patient outcomes.

### Strengths and Limitations

One strength of our study is the use of a large, nationally representative dataset, which enhances the generalizability of our findings. Additionally, the study’s robust methodology, including multivariable logistic regression and model validation, ensures the reliability of the results. However, there are several limitations to consider. The retrospective design may introduce selection bias, as the data were collected from past records, and only patients with complete information were included. The reliance on *ICD-10* codes for identifying conditions and procedures could lead to misclassification or coding errors, potentially affecting the accuracy of our findings. The NIS database lacks detailed clinical information, such as the exact severity of liver cirrhosis or kidney dysfunction, medication use, and procedural specifics, which might limit our ability to account for all potential confounders. Moreover, the database does not provide information on the exact time points of AKI onset or dialysis initiation, which precludes time-to-event analyses and limits temporal interpretation. Additionally, unmeasured variables such as socioeconomic status, lifestyle factors, and detailed laboratory parameters were not available, which could have influenced the outcomes. Finally, while the NIS provides a broad view of inpatient admissions, it does not capture outpatient data, limiting our understanding of long-term outcomes after discharge.

### Conclusions

The results of this study showed that factors independently associated with AKI that requires dialysis following ERCP in patients with liver cirrhosis were decompensated cirrhosis, CKD, obesity, and sepsis. Knowledge of these factors can help enhance ERCP planning and risk stratification and improve patient outcomes in this vulnerable population. Moreover, incorporating detailed information from other databases could enable accurate evaluation and prediction of outcomes in future research.

## Appendix

Multimedia Appendix 1 International Classification of Diseases codes used in the study.

## Abbreviations

AKI: acute kidney injury
aOR: adjusted odds ratio
AUC: area under the curve
CKD: chronic kidney disease
ERCP: endoscopic retrograde cholangiopancreatography
HCUP: Healthcare Cost and Utilization Project
HRS: hepatorenal syndrome
ICD-10: International Classification of Diseases, 10th Edition
NIS: Nationwide Inpatient Sample
OR: odds ratio

## Data Availability

Data are available from the corresponding author upon reasonable request.
