# Supplementary material for: Determinants of Acute Kidney Injury After Endoscopic Retrograde Cholangiopancreatography in Patients With Liver Cirrhosis: Retrospective Observational Study
Source: JMIR Med Inform. 2026 Jul 6;14:e87551. doi: 10.2196/87551 (PMC13386114; doi:10.2196/87551)
Supplement: Multimedia Appendix 1 [file medinform_v14i1e87551_app1.docx]

**Supplementary Table S1. ICD code used in the study**

| Diagnosis or Procedure | ICD-10-CM |
| --- | --- |
| Cirrhosis | K70.3x, K71.7, K74.x |
| Kidney transplantation | Z94.0, T86.10 |
| Chronic hemodialysis | Z49 or procedure codes: 5A1D70Z, 5A1D80Z, 5A1D90Z, 3E1M39Z, and without N17 |
| Acute kidney injury requiring dialysis | N17 with the procedure codes of dialysis:  Z49 or procedure codes: 5A1D70Z, 5A1D80Z, 5A1D90Z, 3E1M39Z |
| Hepatic encephalopathy | K76.82 |
| Portal hypertension | K76.6 |
| Hepatorenal syndrome | K76.7 |
| Esophageal varices | I85.00, I85.01, I85.10, I85.11 |
| Decompensated cirrhosis | K76.82, K76.6, K76.7, I85.00, I85.01, I85.10, I85.11 |
| CKD | I12.0, I13.1, N03.2-N03.7, N05.2-N05.7, N18, N19, N25.0 |
| Obesity | E66.0-E66.2, E66.8, E66.9, Z68.3-Z68.4 |
| Alcohol /drug abuse | F10.0, F10.1x, F10.2x |
| Diabetes mellitus | E10-E14 |
| Coronary artery disease | I20-I25 |
| Atrial fibrillation | I48.2, I48.91 |
| Congestive heart failure | I09.9, I11.0, I13.0, I13.2, I25.5, I42.0, I42.5-I42.9, I43.x, I50.x, P29.0 |
| Chronic pulmonary disease | I27.8, I27.9, J40 -J47, J60-J67,  J68.4, J70.1, J70.3 |
| Rheumatic disease | M05.x, M06.x, M31.5, M32.x-M34.x, M35.1, M35.3, M36.0 |
| Myocardial infarction | I21.x, I22.x, I25.2 |
| Congestive heart failure | I09.9, I11.0, I13.0, I13.2, I25.5, I42.0, I42.5-I42.9, I43.x, I50.x, P29.0 |
| Peripheral vascular disease | I70.x, I71.x, I73.1, I73.8, I73.9, I77.1, I79.0, I79.2, K55.1, K55.8, K55.9, Z95.8, Z95.9 |
| Cerebrovascular disease | G45.x, G46.x, H34.0, I60.x-I69.x |
| Dementia | F00.x-F03.x, F05.1, G30.x, G31.1 |
| Chronic pulmonary disease | I27.8, I27.9, J40.x-J47.x, J60.x-J67.x, J68.4, J70.1, J70.3 |
| Rheumatic disease | M05.x, M06.x, M31.5, M32.x-M34.x, M35.1, M35.3, M36.0 |
| Peptic ulcer disease | K25.x-K28.x |
| Diabetes without chronic complication | E10.0, E10.l, E10.6, E10.8, E10.9, E11.0, E11.1, E11.6, E11.8, E11.9, E12.0, E12.1, E12.6, E12.8, E12.9, E13.0, E13.1, E13.6, E13.8, E13.9,  E14.0, E14.1, E14.6, E14.8, E14.9 |
| Diabetes with chronic complication | E10.2-E10.5, E10.7, E11.2-E11.5, E11.7, E12.2-E12.5, E12.7, E13.2-E13.5, E13.7, E14.2-E14.5, E14.7 |
| Hemiplegia or paraplegia | G04.1, G11.4, G80.1, G80.2, G81.x, G82.x, G83.0-G83.4, G83.9 |
| Moderate or severe renal disease | I12.0, I13.1, N03.2-N03.7, N05.2-N05.7, N18.x, N19.x, N25.0, Z49.0-Z49.2, Z94.0, Z99.2 |
| Any malignancy, including lymphoma and leukemia, except  malignant neoplasm of skin | C00.x-C26.x, C30.x-C34.x, C37.x-C41.x, C43.x, C45.x-C58.x, C60.x-C76.x, C81.x-C85.x, C88.x, C90.x-C97.x |
| Metastatic solid tumor | C77.x-C80.x |
| AIDS | B20.x-B22.x, B24.x |
| Sepsis | R78.81, A41, R65.2, T81.4, T80.2, A42.7, A22.7, B37.7, A26.7, A28.2, A54.86, B00.7, A32.7, A24.1, A39.2, A20.7, A21.7, A48.3 |
| Infection | L00-L08, A00-B99, T81.43, O86.03, Z16 |
| Post-ERCP* pancreatitis | K85.1, K85.9 |
| Post-ERCP* hemorrhage | R58 |
| Perforation | K82.2, K82.A2, K83.2, K22.3, K26.1, K26.2, K26.3 |

*Endoscopic Retrograde Cholangiopancreatography (ERCP)

ICD-10-PCS:

| 0 | F | J | 4,B,D,G | 8 | Z | Z |
| --- | --- | --- | --- | --- | --- | --- |
| 0 | F | B | 5,6 | 8 | Z | X |
| 0 | F | B | 8,9 | 8 | Z | X |
| 0 | F | 7 | 5,6,8,9 | 8 | Z | Z |
| 0 | F | 9 | C | 8 | Z | Z |
| 0 | F | 7 | 5,6,8,9,D | 8 | D | Z |
| 0 | F | C | 5,7,8,9,C,D | 8 | Z | Z |
